# Pulmonary edema: what can it tell us? A pictorial essay

**DOI:** 10.1590/0100-3984.2024.0130-en

**Published:** 2025-09-28

**Authors:** Matheus Marcelino Dias, Eduardo Kaiser Ururahy Nunes Fonseca, Lucas Chagas Aquino, Roberta Castro Linhares, Lavínia Ferreira Dias, Pedro Paulo Teixeira e Silva Torres, Danilo Perussi Bianco, Murilo Marques Silva, Rodrigo Caruso Chate

**Affiliations:** 1 Hospital Israelita Albert Einstein, São Paulo, SP, Brazil

**Keywords:** Diagnostic imaging, Chest radiology, Pulmonary edema, Diagnóstico por imagem, Radiologia do tórax, Edema pulmonar

## Abstract

Pulmonary edema is a very prevalent condition, especially in the intensive care setting, and can have different origins and be caused by different mechanisms. Imaging tests, particularly computed tomography, are sometimes used in the diagnostic investigation of these patients, and may even help to elucidate the etiology. Therefore, it is essential that radiologists understand the dynamic nature of the changes associated with pulmonary edema and be able to recognize diagnostic clues that indicate possible etiologies, in addition to understanding the underlying mechanisms. This illustrated essay provides a concise review of the pathophysiology of pulmonary edema and highlights its main imaging findings, focusing on the clinical features that help to refine the etiological diagnosis.

## Abstract (pt)

O edema pulmonar é uma condição bastante prevalente, sobretudo no contexto de terapia intensiva, ocasionado por diferentes origens e decorrente de mecanismos diversos. Por vezes, os exames de imagem, em particular a tomografia computadorizada, são utilizados na investigação diagnóstica desses pacientes, podendo auxiliar, inclusive, na elucidação etiológica. Portanto, é essencial que os radiologistas entendam a natureza dinâmica das alterações associadas ao edema pulmonar e sejam capazes de reconhecer pistas diagnósticas que indiquem possíveis etiologias, além de compreender os mecanismos subjacentes. Este ensaio ilustrado oferece uma revisão concisa da fisiopatologia do edema pulmonar e destaca seus principais achados em imagens, enfocando as características clínicas que ajudam a refinar o diagnóstico etiológico.

## INTRODUCTION

**Figure 1:**
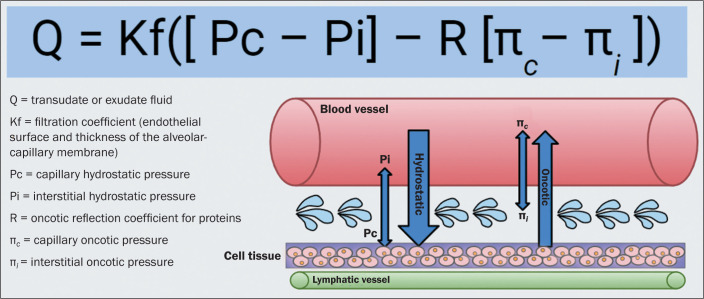
Schematic representation of Starling forces and their actions in the formation of fluid.

Pulmonary edema can be defined as an abnormal accumulation of fluid in the extravascular space within the interstitium and within alveoli^(1)^. It is the result of an imbalance in Starling forces that is not fully corrected by physiological responses^(1,2)^, which include increased lymphatic capacity and decreased interstitial oncotic pressure via the semipermeable barrier known as the alveolar-capillary membrane (Figure 1).

The Starling force imbalance^(2)^ explains the origin of the three main mechanisms of pulmonary edema formation, which facilitate understanding of the various chest imaging findings (Table 1). First, there is increased hydrostatic pressure^(1)^, which leads to a pressure imbalance when hydrostatic pressure exceeds oncotic pressure and fluid accumulates in the lung interstitium, potentially reaching the alveoli. The second mechanism is increased permeability^(1)^, which occurs when a significant inflammatory process causes damage to the capillary endothelium and alveolar epithelium. Finally, there is the mixed mechanism^(1)^, which is a combination of increased hydrostatic pressure and increased permeability^(1)^.

**Table 1:** Differences in imaging findings between the two mechanisms of pulmonary edema formation.

Increased hydrostatic pressure	Increased permeability
Engorgement of the vascular pedicle	Ground-glass opacity (gravitational gradient)
Redistribution of pulmonary blood flow	Consolidation
Loss of definition of segmental vessels	–
Septal lines	–
Pleural effusion	–
Cardiomegaly	–

## PULMONARY EDEMA DUE TO PRESSURE IMBALANCE

**Figure 2:**
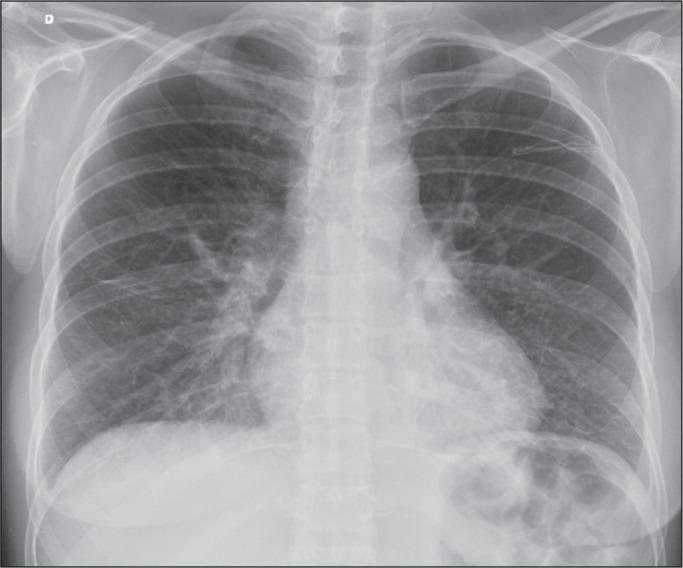
A 32-year-old patient hospitalized for a pulmonary infection. Chest X-ray showing peripheral septal lines.

**Figure 3:**
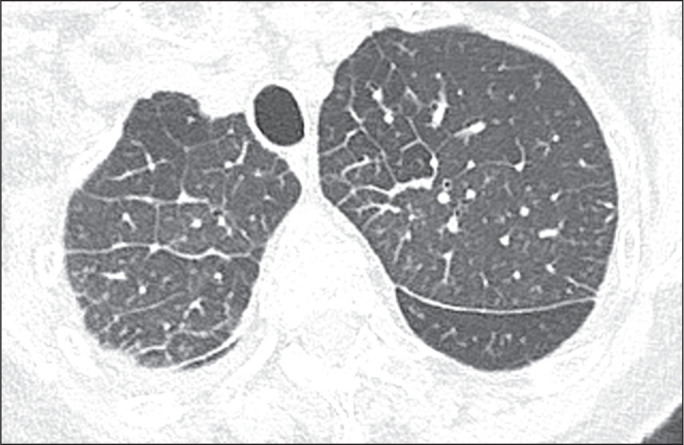
A 53-year-old man with postoperative fluid overload and a pulmonary capillary wedge pressure of 20 mmHg. Computed tomography scan of the chest showing interlobular and intralobular septal lines, peribronchial thickening, and pleural effusion.

Pulmonary edema associated with altered hydrostatic pressure most often results from increased hydrostatic pressure and is primarily caused by heart failure^(2-5)^ or fluid overload^(1)^. It should be noted, however, that there are also cases of negative pressure edema, in which vascular pressure is normal but there is a greater reduction in alveolar pressure, generating a pressure differential that results in fluid migration into the alveolar space. Table 1 shows the imaging findings that corroborate this mechanism^(1,6)^: engorgement of the vascular pedicle with enlargement of the upper portion of the mediastinum—above the aortic arch; cephalization, with redistribution of blood to the upper lobes; engorgement of the bronchovascular bundle caused by increased interstitial thickness around the walls of a bronchus or bronchiole, which, on tomography, appears similar to bronchial wall thickening without luminal narrowing; septal lines^(3)^, previously known as Kerley B lines, which reflect congestion and engorgement of the veins and lymphatic vessels in the interlobular septa, as seen on chest X-rays (Figures 2 and 3); increased density or ground-glass opacities; and, more commonly, pleural effusion and signs of cardiomegaly^(2,3)^.

## ACUTE LUNG EDEMA

**Figure 4:**
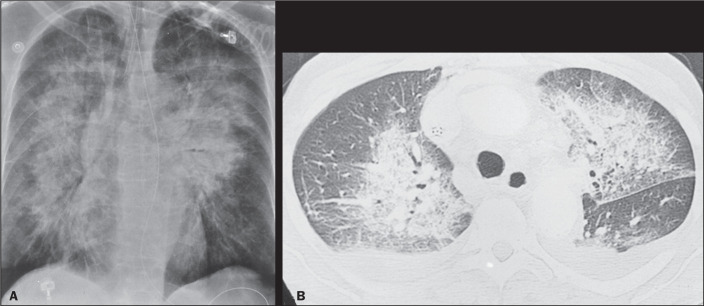
A 71-year-old woman with heart failure. Chest X-ray (**A**) and computed tomography scan (**B**) showing bat-wing alveolar edema with a central distribution.

In cases of a rapid increase in pulmonary pressure, there can be extravasation of fluid into the alveolar space, which tends to occur more commonly in the central regions of the lungs, where the pressure is higher, generating the classic appearance of ground-glass opacities and consolidations in a bat-wing pattern^(1)^, in which the periphery is unaffected (Figure 4).

**Figure 5:**
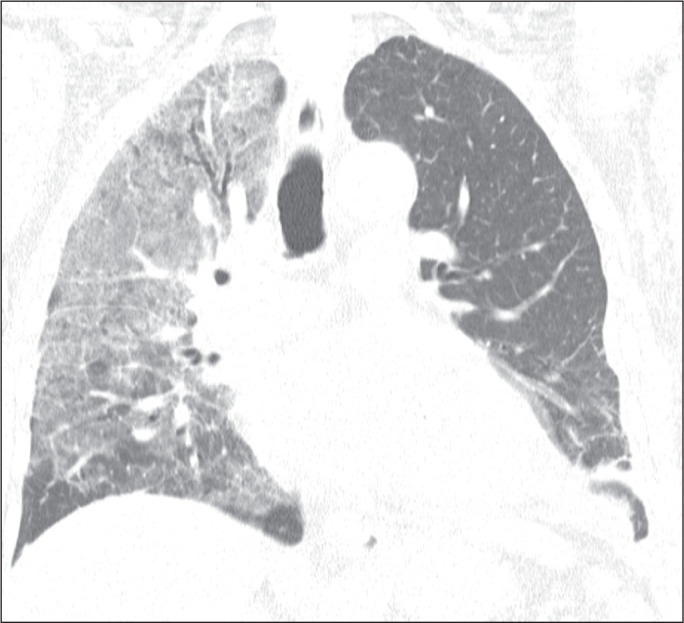
A 77-year-old man hospitalized for acute heart failure after mitral leaflet rupture. Coronal reformatted computed tomography scan of the chest showing extensive ground-glass opacities involving the right upper and middle lobes.

There is another atypical form related to increased hydrostatic pressure: asymmetric pulmonary edema that occurs as a consequence of acute mitral insufficiency^(3)^, resulting in a sudden increase (to 10–15 mmHg) in the left atrial pressure^(1)^, with the regurgitant jet most frequently directed toward the ostium of the right superior pulmonary vein, leading to unilateral edema, which usually affects the upper lobe of the right lung^(1)^, as illustrated in Figure 5.

## NEGATIVE-PRESSURE (POSTOBSTRUCTIVE) PULMONARY EDEMA

**Figure 6:**
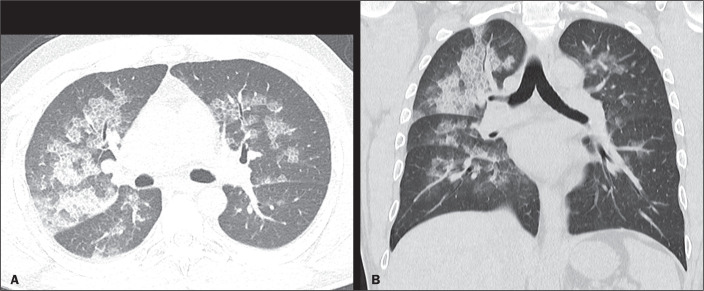
A 44-year-old man with dyspnea after extubation. Computed tomography scans of the chest, with lung window settings, in the axial and coronal planes (**A** and **B**, respectively), showing ground-glass opacities together with interlobular and intralobular septal thickening (the crazy-paving pattern), some converging into foci of alveolar consolidation, predominantly in the central regions, in both lungs.

Negative-pressure pulmonary edema, also known as postobstructive pulmonary edema, occurs after sudden upper airway obstruction in cases of laryngospasm, epiglottitis, strangulation, recent extubation, or seizure. The patient performs forced inspirations, generating abnormally negative intrathoracic pressures (reaching −50 to −100 cmH_2_O), thus increasing venous return to the right heart and elevating hydrostatic pressure in the pulmonary capillaries, favoring fluid extravasation into the interstitium and alveoli, as well as the formation of edema. In such patients, tomography reveals ground-glass opacities, with or without consolidations, predominantly in the central regions of the lungs, with no pleural effusion or smooth septal thickening outside the affected areas (Figure 6).As described by Cascade et al.^(4)^, negative-pressure pulmonary edema resolves rapidly after airway clearance and adequate ventilatory support.

## PULMONARY EDEMA DUE TO INCREASED VASCULAR PERMEABILITY

Edema due to increased vascular permeability arises from changes in the permeability of the alveolar–capillary membrane, and there may or may not be damage to the alveolar epithelium^(1-3)^. The main clinical entity associated with this histological pattern is diffuse alveolar damage^(2,5)^, which increases vascular permeability, causing direct damage to the alveolus. The differential diagnosis should include other etiologies, although only those that do not cause significant alveolar damage, such as high-altitude pulmonary edema^(1)^.

## DIFFUSE ALVEOLAR DAMAGE

**Figure 7:**
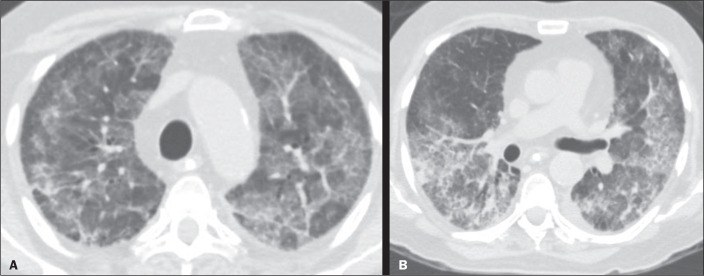
A 47-year-old man who presented with rapidly progressive dyspnea after an airway infection. Computed tomography scan showing diffuse pulmonary opacities, with a consolidative component in the dependent regions and ground-glass opacities in the non-dependent regions. No specific pathogenic agent was identified, and the histopathology demonstrated a pattern of diffuse alveolar damage. The final diagnosis was acute interstitial pneumonia, one of the rare idiopathic interstitial lung diseases.

Diffuse alveolar damage reflects an alteration of the biochemical balance, with an excess of cytokines and interleukins, which increase the permeability of the vascular endothelial plasma membrane, with direct damage to the membrane by inflammatory cells^(1,2)^. The most classic imaging findings in the acute phase include ground-glass opacities and consolidations, typically with a gravitational gradient^(1)^, with denser opacities being noted in the dependent regions of the lung (Figure 7). Some cases progress to fibrosis, typically in the most anterior/non-dependent regions, through a barotrauma/volutrauma mechanism.

## UREMIC PULMONARY EDEMA

**Figure 8:**
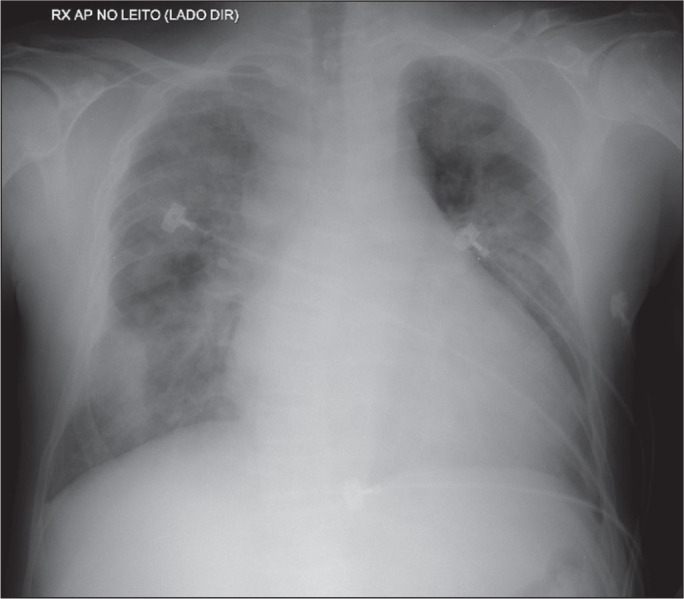
A 78-year-old patient hospitalized for chronic kidney disease requiring dialysis, with an exacerbation that progressed to dyspnea. A bedside chest X-ray showing scattered, poorly defined opacities throughout both hemithoraces. The patient underwent ventilatory support and respiratory therapy, with improvement in the clinical condition and imaging findings after the renal decompensation was controlled.

Pulmonary edema of uremic origin is the result of changes in the pulmonary intravascular Starling forces due to increased permeability of the pulmonary capillary membrane, due to a high protein content in the pulmonary edema fluid. On imaging (Figure 8), sparse foci pulmonary congestion are quite often observed, with an appearance that can resemble bat-wing edema^(7,8)^.

## MIXED-MECHANISM PULMONARY EDEMA

Pulmonary edema due to a mixed mechanism involves changes in hydrostatic pressure and in vascular permeability^(1,2)^, with the main representatives being neurogenic pulmonary edema, pulmonary edema due to reperfusion, pulmonary edema after lung transplantation, pulmonary edema due to lung reexpansion, and postpneumonectomy pulmonary edema^(1,2)^.

## NEUROGENIC PULMONARY EDEMA

**Figure 9:**
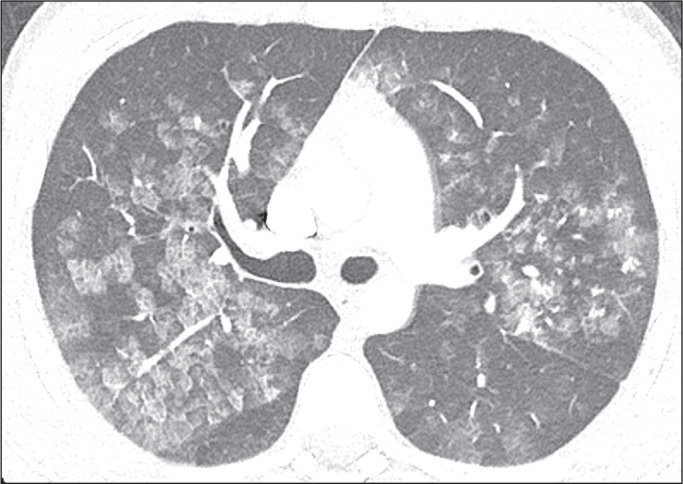
A 20-year-old patient undergoing investigation for dyspnea after a seizure. Computed tomography scan of the chest showing multiple bilateral centrilobular opacities in the lung apices, most of them ground-glass opacities, some of higher density, forming centrilobular nodules. Among the differential diagnoses, neurogenic edema should be considered.

The diagnosis of neurogenic pulmonary edema requires correlation with the clinical history, given that it manifests as acute respiratory distress secondary to severe neurological events^(9)^, such as head trauma, subarachnoid hemorrhage, and seizure^(1,9)^. It is believed to involve autonomic dysfunction with systemic vasoconstriction and release of inflammatory mediators, causing pulmonary hypertension and increasing capillary permeability^(1)^. Imaging shows bilateral consolidations, predominantly at the lung apices (Figure 9), with a heterogeneous pattern. With appropriate treatment of the neurological cause, the thoracic findings usually resolve within 48 h^(1)^.

## PULMONARY EDEMA AFTER LUNG TRANSPLANTATION

**Figure 10:**
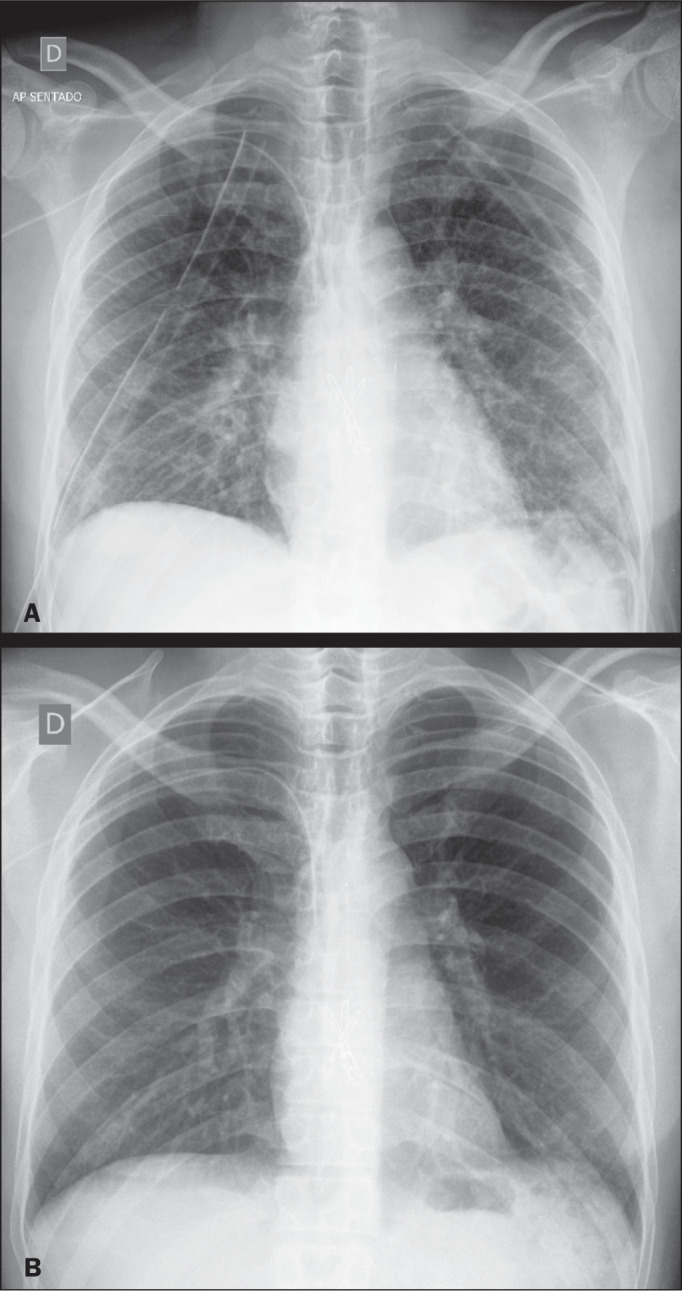
A 34-year-old man with cystic fibrosis who underwent bilateral lung transplantation. A chest X-ray taken 48 h later (**A**) showing accentuated bronchovascular markings, with several septal lines and discrete confluent alveolar opacities. Seven days later, those features had diminished markedly, as shown on a follow-up chest X-ray (**B**). The cardiac and vascular indexes were normal.

Pulmonary edema after lung transplantation involves the permeability mechanism, due to the hypoxia process in the donor lung, which generates ischemia and a loss of surfactant, as well as interrupting surgical lymphatic drainage and surgical denervation^(1)^. The most characteristic imaging findings are perihilar ground-glass opacities, thickening of the peribronchial interstitium, and alveolar hyperdensity in the lower lobes^(1,2)^, as shown in Figure 10, all of which appear 1–7 days after lung transplantation^(1)^.

## PULMONARY EDEMA AFTER NONFATAL DROWNING

**Figure 11:**
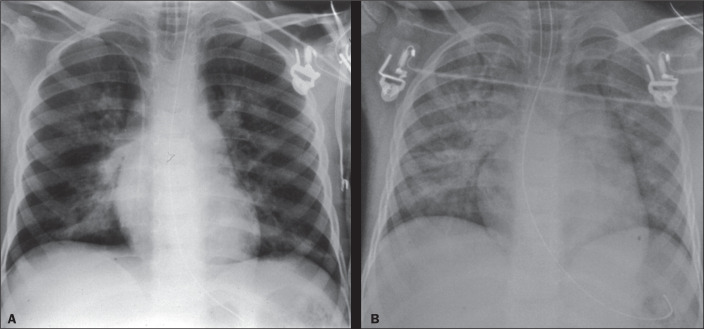
A 5-year-old boy, one hour after experiencing nonfatal drowning in a swimming pool. A chest X-ray obtained at admission (**A**) showing diffuse confluent alveolar patterns of pulmonary edema and peribronchial thickening, with significant improvement seen on another chest X-ray taken three hours after the event (**B**).

**Figure 12:**
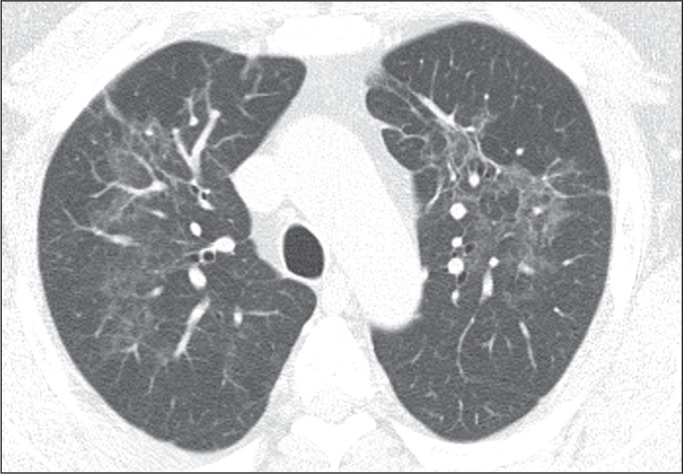
A 3-year-old girl, three hours after experiencing nonfatal drowning in a swimming pool. Computer tomography scan of the chest acquired at that time, showing ground-glass opacities, alveolar opacities, diffuse confluent opacities, and interlobular thickening.

Another uncommon etiology of pulmonary edema is an episode of inhalation, or aspiration, of water (typically during nonfatal drowning) within the preceding 24 h. There are two mechanisms of such edema: negative pressure due to laryngeal spasm; and increased permeability due to alveolar damage triggered by aspirated water coming into contact with the alveoli. As illustrated in Figures 11 and 12, the imaging findings of pulmonary edema after nonfatal drowning are similar to those of other causes of noncardiogenic edema, usually without cardiomegaly or pleural effusion, with a history of nonfatal drowning being essential^(1)^.

## REEXPANSION PULMONARY EDEMA

**Figure 13:**
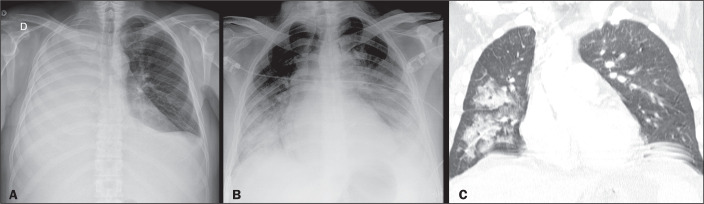
A 57-year-old man hospitalized for pleural carcinomatosis with massive right-sided effusion and an opaque hemithorax, as shown on a chest X-ray obtained at admission (**A**). Three liters of fluid were drained. A follow-up chest X-ray taken two hours later (**B**), showing extensive pulmonary edema on the same side, as was also seen on a computer tomography scan of the chest (**C**). The radiological signs resolved within five days.

Reexpansion pulmonary edema is associated with a history of lung reexpansion after thoracentesis. This type of pulmonary edema typically occurs, via a mixed mechanism, 48 h after the procedure, resolving by postprocedure day 7^(1)^. Typical radiological manifestations include perihilar ground-glass opacities and small central consolidations on the drained, reexpanded side, as well as peribronchial thickening. Common findings include areas of ground-glass opacity and even consolidation^(10,11)^, together with thickening of the bronchovascular bundles and interlobular septal thickening^(1)^, as illustrated in Figure 13.

## HIGH-ALTITUDE PULMONARY EDEMA

**Figure 14:**
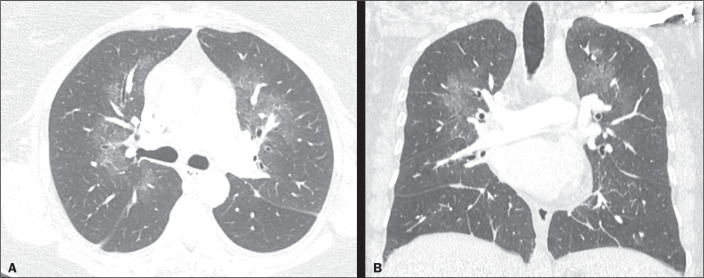
A 51-year-old patient with a history of travel to Peru, including a visit to the Rainbow Mountains at an altitude of 5,200 m, who, upon returning to Brazil, presented with flu-like symptoms and bloody sputum. Computed tomography scans of the chest in the axial and coronal planes (left and right panels, respectively), showing thickening of the bronchial walls and interlobular pulmonary septa, together with ground-glass opacities are observed, predominantly superior and perihilar, in both lungs, suggestive of high-altitude pulmonary edema.

The epidemiological history also plays a very important role in the diagnosis of high-altitude pulmonary edema, given that the condition is directly related to exposure to low oxygenation, typically occurring within three days after an ascent to an altitude above 3,000 m^(1)^. The mechanism is classified as mixed, due to transient sympathetic discharge causing pulmonary vasoconstriction that leads to pressure imbalance and changes in the transmembrane potential^(1)^, as depicted in Figure 14.

## LUNG ULTRASOUND

Lung ultrasound, a noninvasive, radiation-free examination, is especially useful in intensive care units because it allows rapid differentiation among the various types of pulmonary edema. The presence of septal lines—reverberation artifacts originating from the hyperechoic pleural line—allows for semiquantitative estimation of extravascular lung water content, ranging from normal to indicative of severe edema^(12,13)^. Bilateral pleural-line abnormalities, such as thickening, irregularity, or fragmentation, are suggestive of noncardiogenic edema^(14)^. Surface wave elastography can be employed to assess the elastic properties of lung tissue, contributing to the characterization of edema^(13)^.

## CONCLUSION

Pulmonary edema, which reflects the leakage of fluid into the interstitium and alveolar spaces, has several underlying mechanisms. Imaging, combined with clinical history, can lead to a diagnosis in challenging cases and can allow timely follow-up of affected patients. Therefore, radiologists must understand the mechanisms and key imaging aspects involved.

## Data availability statement.

Not applicable.
